# Supplementary material for: Accumulation of Fat Not Responsible for Femoral Head Necrosis, Revealed by Single-Cell RNA Sequencing: A Preliminary Study
Source: Biomolecules. 2023 Jan 13;13(1):171. doi: 10.3390/biom13010171 (PMC9856115; doi:10.3390/biom13010171)
Supplement: Supplementary file 1 [file biomolecules-13-00171-s001.zip › biomolecules-2035360-supplementary.pdf]

biomolecules

MDPI

Supplementary Materials

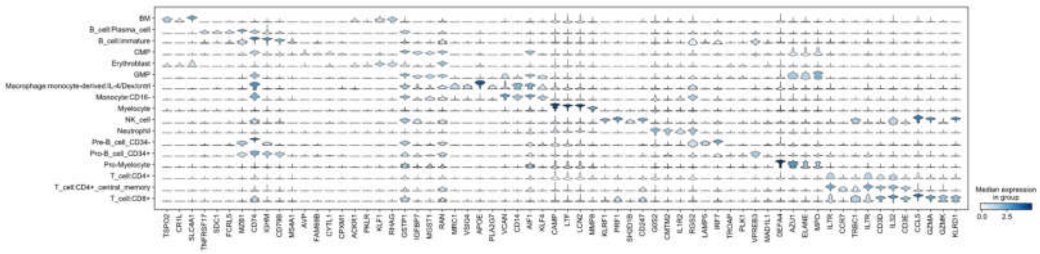

Figure S1. Marker genes defining 17 cell types.

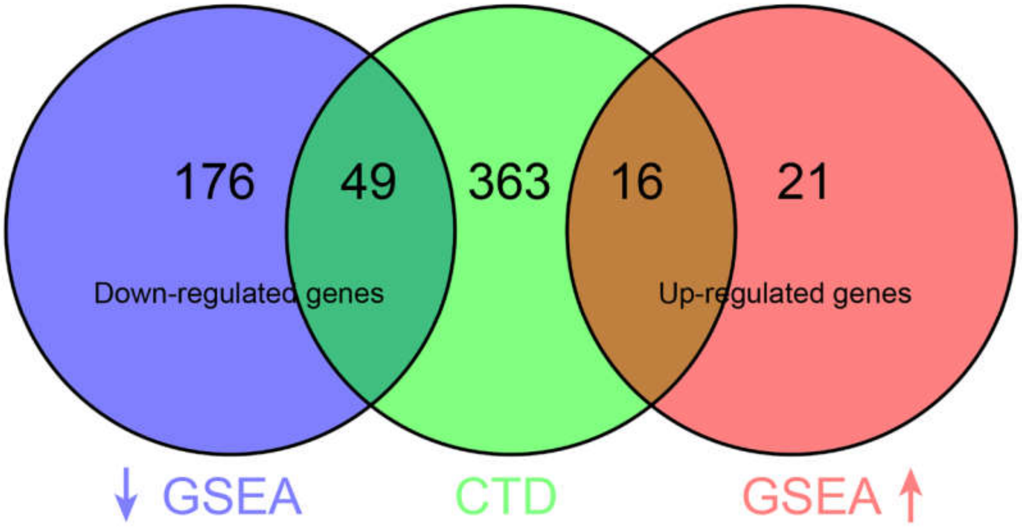

Figure S2. 65 common targeted genes of glucocorticoid, including 16 upregulated genes and 49 down-regulated genes, obtained from the intersection of Comparative Toxicogenomics Database (CTD) and Gene Set Enrichment Analysis (GSEA) Database.

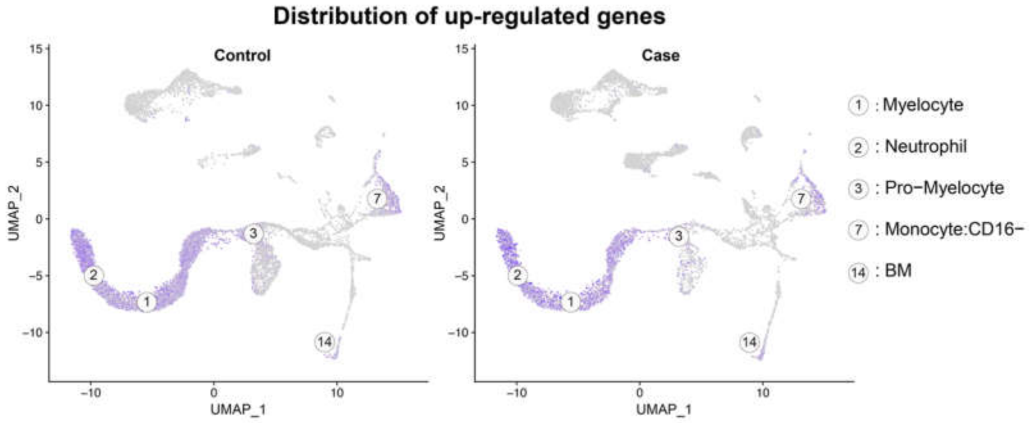

Figure S3. The distribution of 16 glucocorticoid-induced up-regulated genes in control and case group.

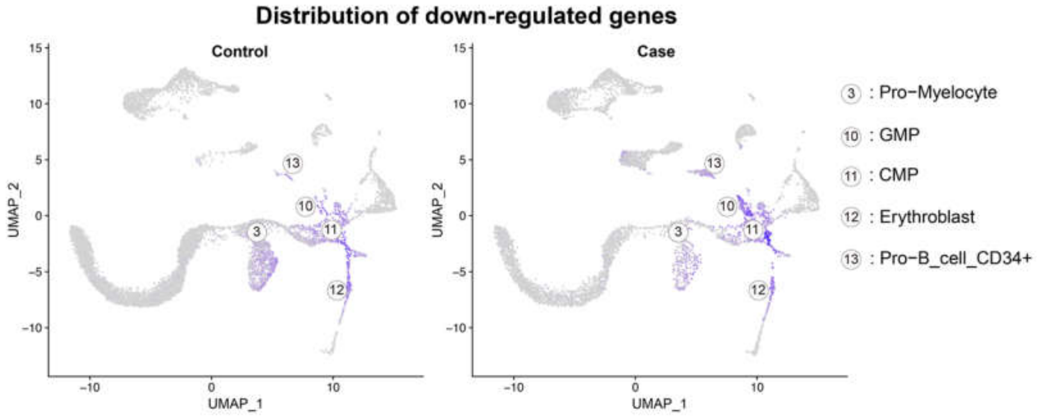

Figure S4. The distribution of 49 glucocorticoid-induced down-regulated genes in control and case group.

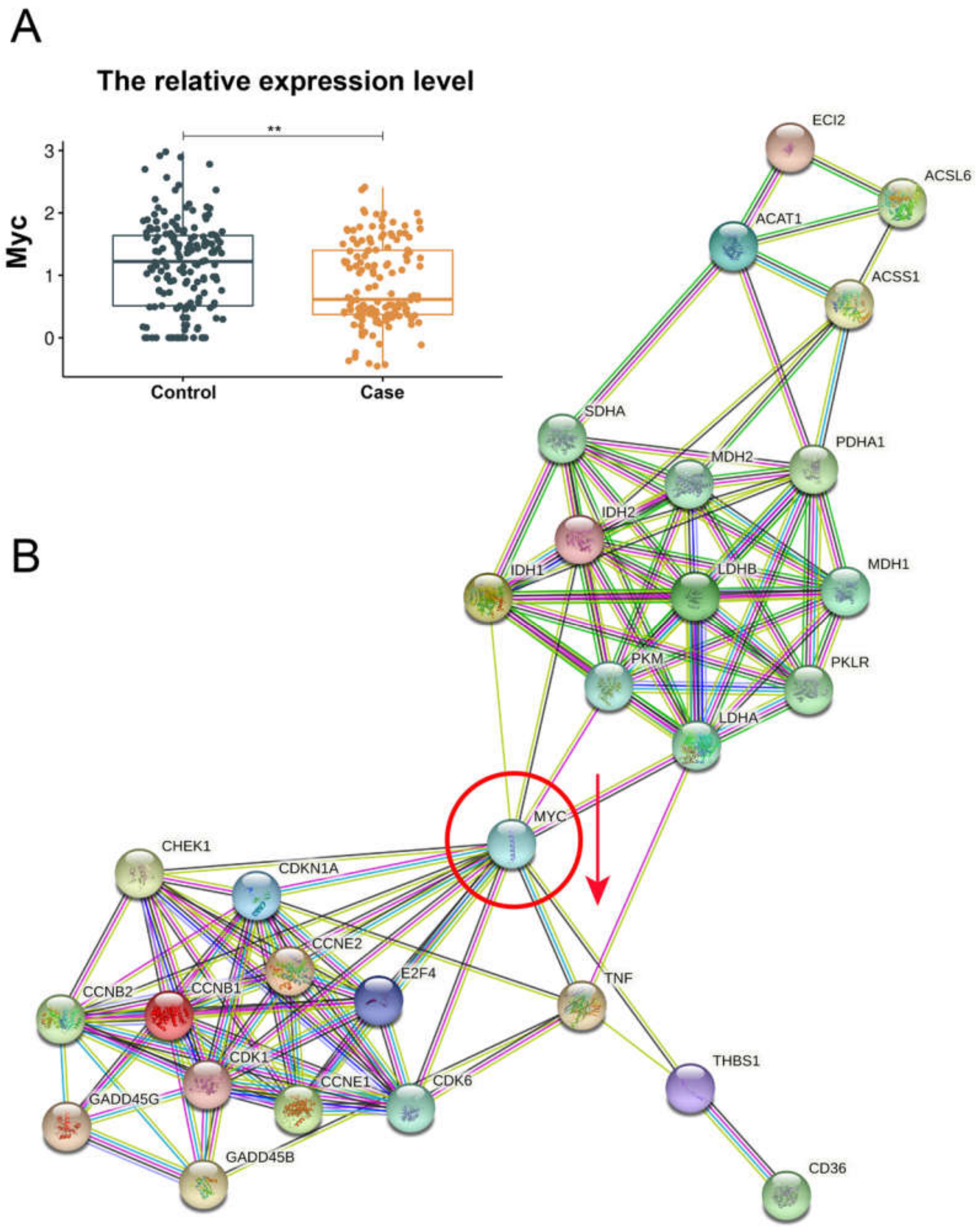

Figure S5. The network of 29 differentially expressed genes in erythroblast. The relative lower expression level of *Myc* in the case group compared with that in the control group (**A**). The protein-protein interaction network of 29 differentially expressed genes in erythroblast, and every gene plays a role in at least two signaling pathways of glucose and fat metabolism correlative (**B**). *Myc* serves as a network hub.

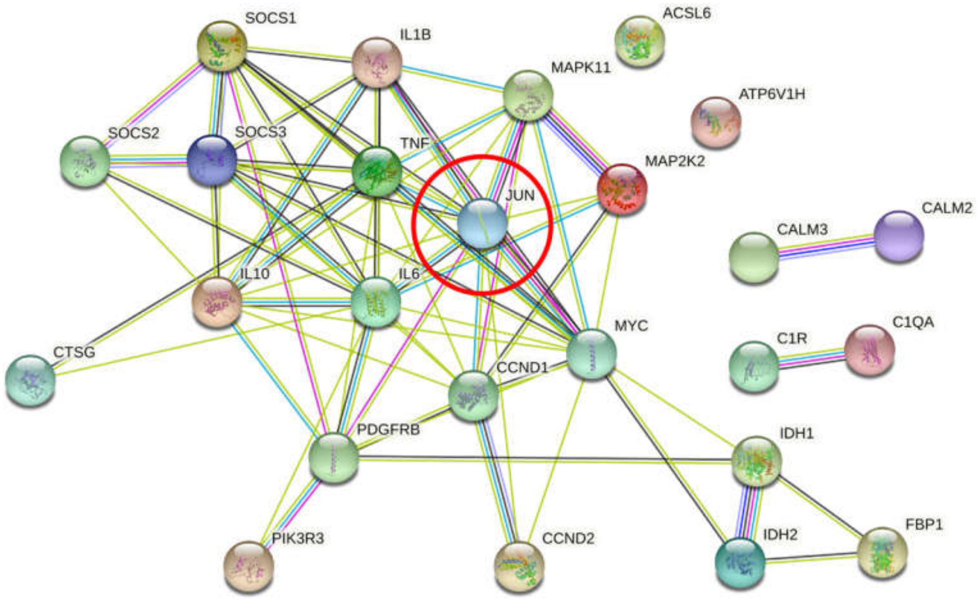

Figure S6. The protein-protein interaction of 25 differentially expressed genes in monocyte derived macrophages. Every gene plays a role in at least two inflammation response related signaling pathways. *Jun* serves as the network hub of the inflammatory response.

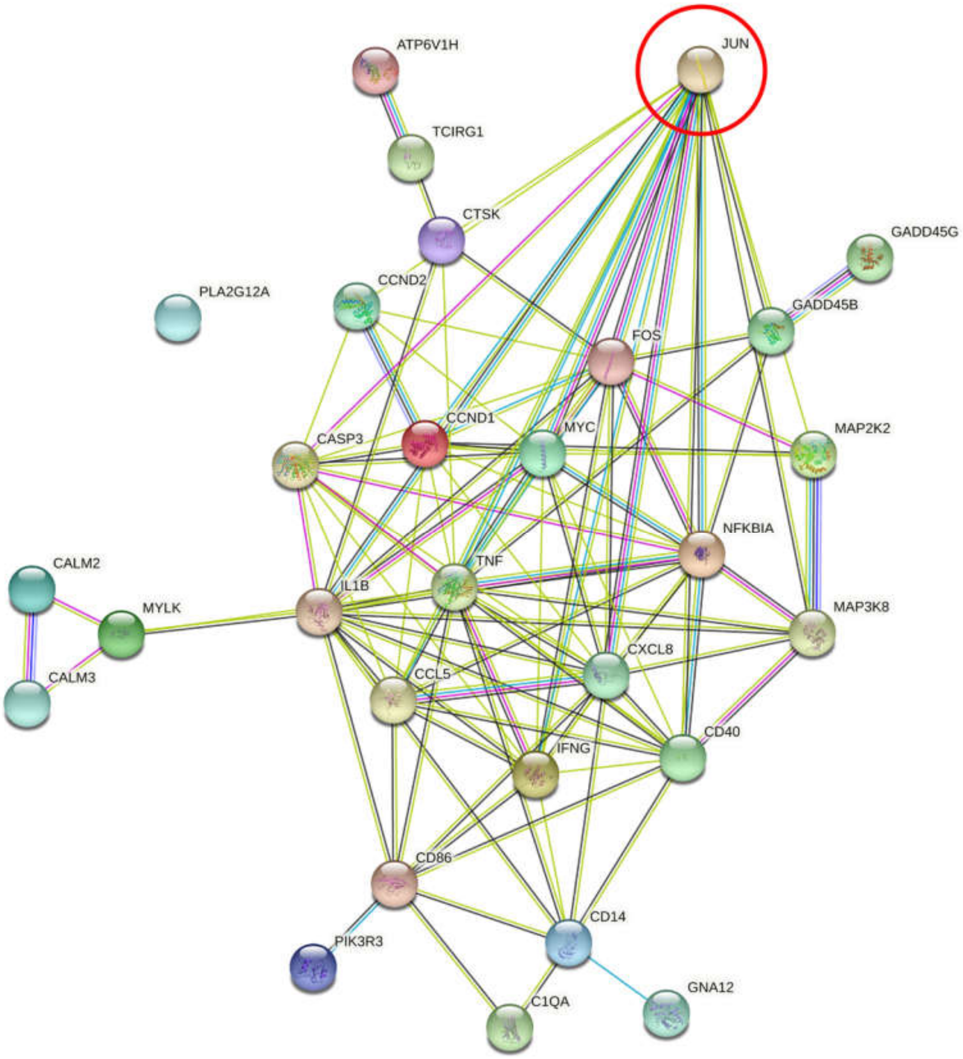

Figure S7. The protein-protein interaction of 29 differentially expressed genes in immature B cell. Every gene plays a role in at least two blood vessel and bone metabolism related signaling pathways, and *Jun* serves as a network hub.

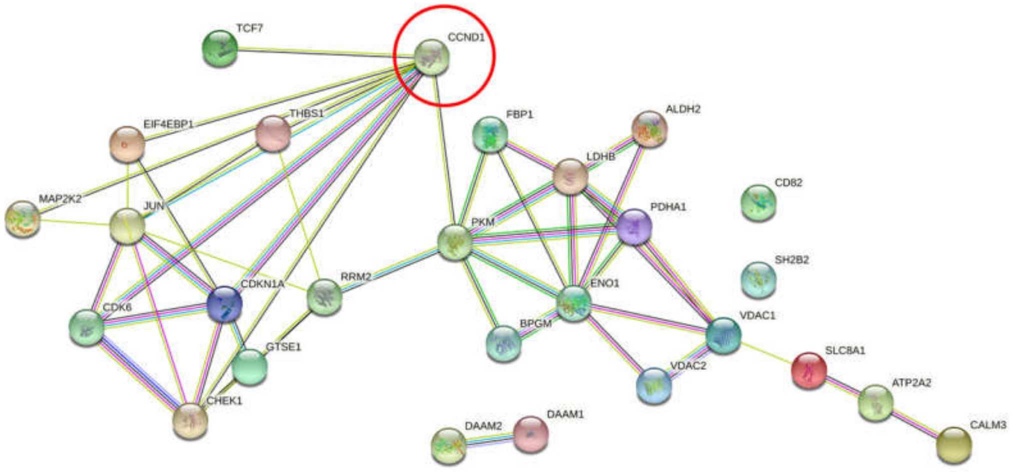

Figure S8. The protein-protein interaction of 27 differentially expressed genes in neutrophil. Every gene plays a role in blood vessel and bone metabolism related signaling pathways and *Ccnd1* served as the network hub.

Table S1. 65 glucocorticoid-associated dysregulated genes obtained from the intersection of Comparative Toxicogenomics Database and Gene Set Enrichment Analysis Database.

BCL2	NAP1L1	RGS2	CD99	NASP	RGCC
LDHB	PDLIM1	HSP90AB1	P2RX5	SET	NPM1
JUN	PPIA	AREG	SNRPD3	LYZ	IL1R2
ANP32B	LTF	BIRC3	OLA1	ETFA	EZR
SSBP2	MAP3K8	HSPA8	SSBP1	RPLP0	PRDX6
BLNK	AHNAK	GAPDH	TUFM	PRDX4	VDAC1
CSTA	AKAP12	HSPB1	SERBP1	ALAS2	STMN1
MARCKSL1	CD9	DEFA4	ST13	TUBA1B	IDH2
C1QBP	FABP5	DEFA1	PSIP1	PDIA6	PRDX1
DYNLL1	G0S2	APOBEC3G	CCT8	DUT	CXCL8
PRMT1	FOSB	STOML2	HMGN2	PARK7	
